# Expression of dNK cells and their cytokines in twin pregnancies with preeclampsia

**DOI:** 10.6061/clinics/2019/e1200

**Published:** 2019-10-30

**Authors:** Isabela K.R. Agra, Adolfo W. Liao, Mara S. Hoshida, Regina Schultz, Marcello P. Toscano, Rossana P.V. Francisco, Marcelo Zugaib, Maria L. Brizot

**Affiliations:** I Departamento de Ginecologia e Obstetricia, Hospital das Clinicas HCFMUSP, Faculdade de Medicina, Universidade de Sao Paulo, Sao Paulo, SP, BR.; II Departamento de Patologia, Faculdade de Medicina FMUSP, Universidade de Sao Paulo, Sao Paulo, SP, BR.

**Keywords:** Twin Pregnancy, Preeclampsia, Decidual Natural Killer Cells, Interleukins

## Abstract

**OBJECTIVES:**

To assess the expression of decidual natural killer (dNK) cells and their cytokines in twin pregnancies with preeclampsia.

**METHODS:**

This was a prospective case-control study. The inclusion criteria were diamniotic (monochorionic or dichorionic) twin pregnancies in the third trimester with negative serological results for infectious diseases; absence of major fetal abnormalities or twin-twin transfusion syndrome; and no history of administration of corticosteroids in this pregnancy. The control group (CG) included uncomplicated twin pregnancies, and the preeclampsia group (PEG) included twin gestations with clinical and laboratory confirmation of the disease according to well-established criteria. Samples of the decidua were obtained and analyzed by immunohistochemistry for the expression of dNK cells and interleukins (ILs) 10, 12 and 15. In addition, maternal serum samples were collected to determine the levels of these interleukins.

**RESULTS:**

Thirty twin pregnancies were selected: 20 in the control group (CG) and 10 in the preeclampsia group (PEG). The PEG showed strong placental immunostaining for IL-15 (*p*=0.001) and high maternal serum levels of IL-10 (22.7 *vs*. 11.9 pg/mL, *p*=0.024) and IL-15 (15.9 *vs*. 7.4 pg/mL, *p*=0.024).

**CONCLUSION:**

A higher maternal serum concentration of both pro- and anti-inflammatory factors was observed in the twin pregnancies in the PEG. However, no difference in placental expression of IL-10 was found between the groups. These findings may suggest that maternal attempts to balance these interleukins were not sufficient to cause a placental response, and this failure may contribute to the development of preeclampsia.

## INTRODUCTION

Preeclampsia (PE) is the leading cause of maternal and perinatal morbidity and mortality worldwide (1,2). Although the underlying pathophysiology of PE remains partially unknown, relevant characteristics of this disease include maternal endothelial dysfunction and inflammatory imbalance; most recently, chronic immune activation was also noted (3-5). Importantly, the definitive solution for PE is the delivery of the placenta (6), leading to investigations related to the dynamic interactions involving the maternal-fetal interface.

Interest has been focused on the role of decidual natural killer (dNK) cells, not only for their numerical importance as one of the main cell types present in early maternal decidua but also because of their unique functional properties, which may represent novel immunotherapeutic opportunities (7,8). Controversy still exists regarding the behavior of dNK cells in PE: whereas some studies have demonstrated higher concentrations of this cell type (9,10), others have observed lower expression of dNK cells in singleton gestations with PE (11-13).

Additionally, several studies have investigated the profiles of dNK regulatory cytokines in the pathophysiology of PE. These substances are produced in the maternal-fetal interface and are responsible for complex interactions between the distinct cell types present at the decidua, contributing to trophoblastic invasion (14). Among these cytokines, there is strong evidence in the literature of the crucial role of interleukins (IL) 10, 12 and 15 as important regulators of dNK cell functions (5,8).

Patients with PE tend toward an inflammatory profile, as demonstrated by increased maternal circulating levels of IL-15 (10,15). Hromadnikova et al. recently demonstrated that this interleukin could increase the production of interferon-gamma (IFN-γ), a substance with known deleterious effects on trophoblastic invasion (16). In addition, IFN-γ acts on dNK cells by means of a feedback mechanism, promoting the production and release of IL-12, which is the main dNK cell stimulating factor (17). Therefore, this cascade may perpetuate a harmful inflammatory cycle.

Furthermore, some authors have observed lower placental expression of IL-10 in cases of PE (9,18). This cytokine plays an important anti-inflammatory role and may prevent potential obstruction of angiogenesis and trophoblastic invasion, thus protecting against PE development (19). In addition to the protective role of IL-10 in inflammation, Kalkunte et al. (20) proposed that IL-10 may function as a vital bridge that links immunity, placental angiogenesis, and hypoxia at the maternal-fetal interface. However, the data relating to maternal circulating levels of IL-10 in PE remain inconclusive (21,22).

Notably, however, all these studies were performed in singleton pregnancies, and in our literature search, we did not find studies investigating dNK cells in twin pregnancies with PE. Considering that the incidence of PE is higher in twin gestations and occurs in more severe forms in this group (23), we sought to investigate the pathophysiology of this disease in twin pregnancies through observation of dNK cells and their regulatory cytokines.

## MATERIALS AND METHODS

### Study Design

This was a prospective case-control study, conducted in the Multiple Pregnancy Unit of Department of Obstetrics and Gynecology of São Paulo University Medical School, from July 2015 to June 2017. The study protocol was approved by the University Ethical Committee (46741815.8.0000.0068).

The inclusion criteria used were as follows: diamniotic (monochorionic or dichorionic) twin pregnancies in the third trimester with negative serological results for infectious diseases; absence of major fetal abnormalities or twin-twin transfusion syndrome; and no history of administration of corticosteroids in this pregnancy. The exclusion criteria used were rupture of amniotic membranes for a prolonged time (greater than 12 hours); failure to obtain fragments of placenta decidua after delivery; failure to obtain laboratory results; or postnatal diagnosis of congenital anomaly or genetic syndrome in at least one of the newborns.

The control group (CG) included uncomplicated twin pregnancies without any previous or current clinical conditions. The preeclampsia group (PEG) included twin gestations with no other previous or current clinical conditions, except clinical and laboratory confirmation of preeclampsia according to well-established criteria (1): systolic blood pressure of 140 mmHg or more or diastolic blood pressure of 90 mmHg or more on two occasions, at least 4 hours apart, after 20 weeks of gestation in a woman with a previously normal blood pressure, associated with proteinuria (300 mg or more per 24 hour urine collection). In the absence of proteinuria, new-onset hypertension with thrombocytopenia, renal insufficiency, impaired liver function, pulmonary edema, new-onset headache or visual symptoms also indicated PE (1).

### Calculation of Sample Size

Since we were not able to find any studies in the literature describing the expression of dNK cells or their regulatory interleukins in twin pregnancies with PE, the calculation of sample size was based on the study of Olusi et al. (14). In this study, the authors determined the maternal serum concentrations of interleukins in singleton pregnancies with and without PE. The values for IL-10 were PE group: 93.2±24.1 pg/mL; Control group: 31.1±7.0 pg/mL. Assuming the same difference observed by Olusi et al., we would require five patients in each group (significant level, 5%; power, 80%). In order to reduce statistical bias, the number of cases used was duplicated. The patients were then matched by chorionicity in a proportion of two control cases for each case in the study group. For all experiments all cases and controls were considered.

### Sample Collection

Up to seven days before delivery, 10 mL of maternal blood sample was collected by peripheral venous puncture and placed in a tube with EDTA (ethylenediaminetetraacetic acid) and in a dry tube, which were promptly sent to the laboratory for processing: serum and plasma were obtained by centrifugation and stored at -80°C until further analysis.

Placental tissue was taken immediately after delivery, and slices of the maternal decidual region were obtained, fixed in formalin, and paraffin embedded. The placental paraffin-embedded sections (5.0 µm) were deparaffinized in xylene and rehydrated in increasing concentrations of alcohol and distilled water. For heat-induced antigenic retrieval, the sections were placed in 10 mM boiling citrate buffer (pH 6.0) in an electric cooker for 30 min and allowed to cool for 15 min at room temperature (RT). Slides were rinsed in running tap water and 3 changes of phosphate buffered saline (PBS) for 5 min each. Endogenous peroxidase was blocked by incubating the slides with 3 changes of 3% H_2_O_2_ for 20 min each at RT in the dark. The placental tissues were incubated with primary antibodies anti-CD56 (cluster of differentiation of dNK cells clone RNL-1; Abcam, Cambridge, UK; dilution of 1:75), anti-IL-10 (Abcam, Cambridge, UK; dilution of 1:200), anti-IL-12p40 (Abcam, Cambridge, UK; dilution of 1:150) and anti-IL-15 (Abcam, Cambridge, UK; dilution of 1:300) overnight at 4°C. The primary antibody was washed off, and the slides were rinsed 3 times in PBS and then incubated with the secondary antibody (Novolink Polymer, Leica Biosystems Newcastle Ltd.). After rinsing in PBS, the reactions were developed by using the substrate-chromogen DAB (Sigma Chemical Corporation, St. Louis, Missouri, USA). All incubations were performed in a humidity chamber. Placental samples from the CG and PEG were processed simultaneously. The nuclei were counterstained with hematoxylin, and the slides were dehydrated in a series of ethanol dilutions and washed in xylene before putting on coverslips with Tissue Tek Glas Mounting Media (Sakura Finetek, Netherlands). For negative controls, the primary antibody was omitted.

### Morphometric Placental Analysis

Morphometric analyses of the placentas were performed as previously described (24). Briefly, the decidual space was described by analyzing the hematoxylin-stained sections of the placenta. For each section, five areas of the decidual region were randomly selected for the image acquisition (400x magnification) by using a Leica microscope (Leica Biosystems, Germany) and LAS image acquisition software. The images were analyzed using the image processing and analysis program ImageJ (NIH, Bethesda, MA, USA). The images were given a color threshold to cover the area corresponding to the decidual region. The percentage of coverage for each antibody was calculated as the ratio between the number of pixels covered by the area defined by the threshold and the total number of pixels in the image, multiplied by 100, and compared between the groups.

### Serum Analysis

Serum was analyzed in duplicates for IL-10, IL-12 and IL-15 by means of a commercial Milliplex^®^ kit using Luminex^®^ xMAP^®^ technology from EMD Millipore (Merck Millipore Co., Germany).

### Statistical Analysis

Data were analyzed using SPSS software (IBM SPSS Statistics for Windows, Version 20.0. IBM Corp., Armonk, NY). Baseline characteristics described were related to maternal characteristics (age, color, educational level, body mass index and habits), pregnancy and childbirth aspects (parity, blood pressure levels, mode of delivery, time of membrane rupture and preeclampsia specific tests) and neonatal variables (sex, birthweight and Apgar index), followed by immunohistochemical and maternal serum results. Continuous data were expressed as the median. To perform comparisons between groups, the Mann-Whitney U-test was used for quantitative variables, and Fisher’s exact test was used for qualitative variables. A value of *p*<0.05 was considered significant.

## RESULTS

During the study period, 34 eligible pregnant women with twin pregnancies consented to participate: 24 with uncomplicated gestations and 10 with confirmed PE diagnoses. Four patients in the CG were excluded because fragments of placental decidua could not be obtained after delivery. Therefore, 30 pregnant women remained: 20 in the CG (14 dichorionic – DC – and six monochorionic – MC) and 10 in the PEG (7 DC and 3 MC).

The baseline characteristics were similar between the two groups (Table 1). Although a higher proportion of patients defined as non-white (60% *vs*. 35%) and nulliparous (80% *vs*. 40%) were present in the PEG, the difference was not statistically significant (*p*=0.255 and 0.058, respectively).

In the PEG, 70% of the cases were classified as severe due to blood pressure >160/110 mmHg on more than one occasion, according to the American College of Obstetricians and Gynecologists (ACOG) criteria (1). Among those cases, we observed only one event of placental abruption at 31 weeks. Additionally, no cases of hemolysis, elevated liver enzymes, and low platelet count (HELLP) syndrome were diagnosed in the PEG.

**Figure 1:**
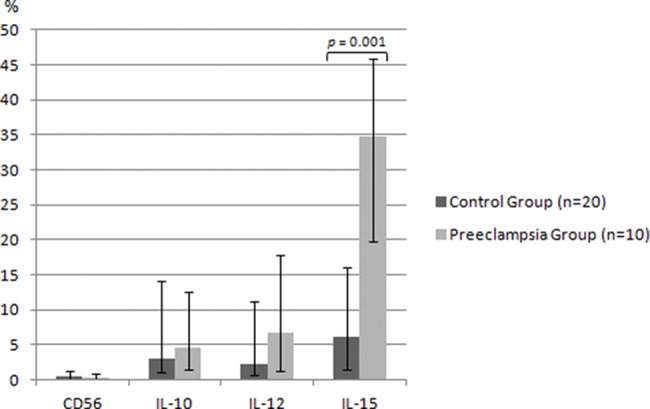
Placental expression of dNK cells and their regulatory interleukins. Percentage of stained area delimited by morphometric analysis. Data described as median (range). CD56 = cluster of differentiation of dNK cells. Mann-Whitney test, *p*<0.05.

**Figure 2:**
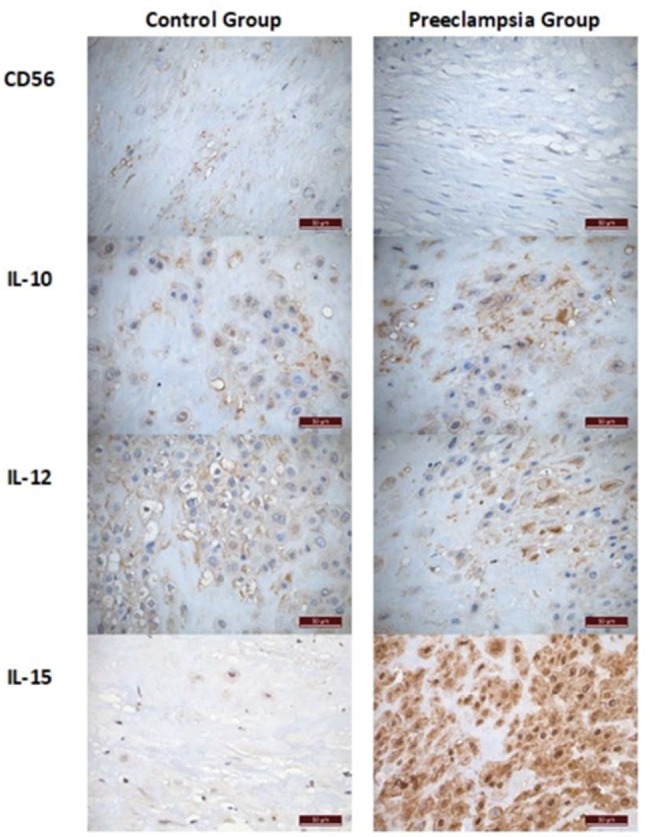
Immunohistochemical expression of dNK cells and their regulatory interleukins in placental decidua. Antibodies anti-CD56, anti-IL-10, anti-IL-12p40 and anti-IL-15 were used. One representative case of each group is presented.

In the immunohistochemical analysis, we observed higher expression of IL-15 in the PEG – 34.82% (19.07-47.37) *versus* 6.09% (1.62-16.33), *p*=0.001 – with no significant difference between the groups with respect to the placental expression of dNK cells or the other factors: for CD56 expression, 0.11% (0.04-0.35) in the PEG *vs*. 0.15% (0.04-0.56) in the CG, *p*=0.231; for IL-10, 4.55% (1.87-12.0) in the PEG *vs*. 3.10% (1.12-14.82) in the CG, *p*=0.231, and for IL-12, 6.76% (1.66-17.59) in the PEG *vs*. 2.17% (0.81-11.57) in the CG (Figure 1). Additionally, we repeated the analysis after removing cases with neonatal birthweight <10^th^ percentile (four in the CG and one in the PEG). The results remained unchanged, with a significant difference between the groups found only for the decidual expression of IL-15 (6.09 *vs*. 37.62, *p*=0.001). Figure 2 compares the morphometric immunohistochemical analyses of these groups.

**Figure 3:**
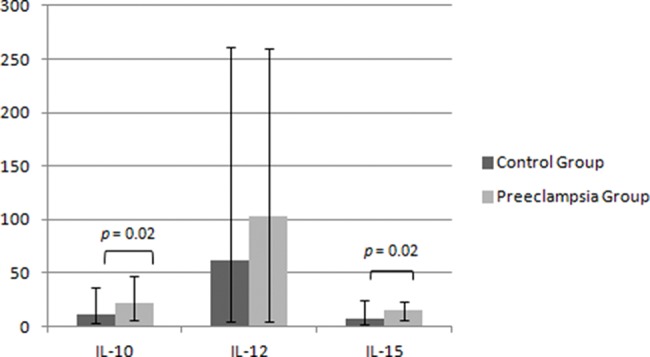
Maternal serum levels of regulatory dNK interleukins. Data described as the median (range), values in pg/ml. IL = interleukin. Mann-Whitney test, *p*<0.05.

The maternal serum analysis demonstrated higher levels of IL-10 and IL-15 in the PEG – 22.7 (4.6-46.4 pg/mL) *vs*. 11.9 (2.1-35.5 pg/mL) for IL-10 and 15.9 (4.0-24.2 pg/mL) *vs*. 7.4 (0.8-25.8 pg/mL) for IL-15, *p*=0.024 – and no significant difference between the two groups for the expression of IL-12 – 102.5 (2.8-251.8 pg/mL) in the PEG *vs*. 61.5 (2.8-253.2 pg/mL) in the CG, *p*=0.373 (Figure 3). Similarly, we repeated the analysis after removing cases with neonatal birthweight <10^th^ percentile and demonstrated the same statistical findings for IL-10 (10.7 *vs* 22.1, *p*=0.017), IL-12 (88.3 *vs*. 73.1, *p*=0.487) and IL-15 (7.5 *vs*. 15.0, *p*=0.027).

## DISCUSSION

The findings of the present study show that twin pregnancies with PE had higher placental expression of IL-15 and higher maternal serum levels of IL-10 and IL-15 than those in the CG.

Although the complete pathophysiology of PE has not yet been elucidated, it is well known that the maternal-fetal placental interface plays an important role (5). Therefore, attention should be concentrated on the predominant cell types present in this region, such as dNK cells. However, even in singleton gestations, there is still no consensus in the literature concerning dNK decidual expression in PE (9,11,25). Consistent with the results demonstrated by Eide et al. (13), in the present study, we did not observe a difference in dNK placental expression between the groups. These findings may suggest a greater physiological importance of dNK cell function and molecular interaction rather than their numerical expression.

Concerning dNK regulatory cytokines, we observed higher maternal serum levels of both anti- and pro-inflammatory interleukins (IL-10 and IL-15, respectively) in the PEG. However, this balance was not sustained in the placental histological analysis; we noticed higher expression of the inflammatory IL-15 in the PEG and no difference between the groups in decidual expression of IL-10 and IL-12. In contrast, the only previous study involving placental analysis of IL-15 in PE demonstrated unchanged levels of this factor in patients with the disease (10). For the other factors, a few studies have observed lower decidual expression of IL-10 and IL-12 in singleton pregnancies with PE (9,10,18).

PE is associated with chronic immune activation, leading to elevated serum levels of inflammatory cytokines. These increased levels of IL-15 may contribute to endothelial dysfunction during PE (5). Additionally, IL-15 may stimulate the production of IFN-γ, a substance with deleterious effects on trophoblastic invasion (26).

Considering PE as a pro-inflammatory state, we can hypothesize that our findings of higher serum levels of both IL-10 and IL-15 may correspond to a maternal response represented by anti-inflammatory feedback. In addition, the absence of a significant difference between the groups in placental expression of IL-10 suggests that the maternal effort to balance pro- and anti-inflammatory responses occurs only at the serum level, with no impact in the decidual region, which may contribute to the development of the disease in PEG.

The main limitation of this study was the small number of cases, which was mainly related to the single-center nature of the analysis and the inclusion of only pure PE cases; therefore, multicenter studies may be necessary to confirm our findings. Notwithstanding, this was the first study that specifically analyzed the expression of dNK cells and their regulatory interleukins in twin pregnancies with PE. Furthermore, our findings may provide insights into possible immune mechanisms involved in the pathophysiology of PE.

## AUTHOR CONTRIBUTIONS

Agra IKR was responsible for the data management/analysis and manuscript writing. Liao AW was responsible for the data analysis and manuscript writing. Hoshida MS, Shultz R and Toscano MP were responsible for the data management and manuscript writing. Francisco RPV and Zugaib M were responsible for the project development and manuscript editing. Brizot ML was responsible for the project development, data management/analysis and manuscript writing.

## Figures and Tables

**Table 1:** Baseline characteristics of study population.

	Control Group (n=20)	Preeclampsia Group (n=10)	*p*
**Maternal characteristics**			
Age, years^a^	30 (19-40)	25 (16-48)	0.328
Non-white, n (%)^b^	7 (35)	6 (60)	0.255
Educational level, years^a^	11 (6-14)	8 (6-14)	0.502
BMI, kg/m^2^ a	27.2 (21.6-47.3)	30.7 (22.0-33.5)	0.397
Smoking, n (%)^b^	3 (15)	0 (0)	0.532
**Pregnancy characteristics**			
Nulliparous, n (%)^b^	8 (40)	8 (80)	0.058
Spontaneous gestation, n (%)^b^	17 (85)	8 (80)	0.999
24h proteinuria, grams	NA	1.07 (0.32-7.9)	NA
Mean BP, mmHg^a^	79.1 (68-93.3)	126.6 (106.6-140)	0.0001
GA PE diagnosis, weeks	NA	34.3 (30.1-37.0)	NA
GA maternal blood sample, weeks^a^	36.9 (35.1-37.5)	36.5 (31.0-37.0)	0.186
GA at delivery, weeks^a^	37.4 (35.1-38.0)	36.7 (31.7-37.2)	0.131
Delivery by cesarean, n (%)^b^	13 (65)	8 (80)	0.675
Time of membrane rupture, hours^a^	5.7 (4-8)	8.0 (4-12)	0.423
Placental total weight, grams^a^	852.5 (515-1278)	755.0 (570-1030)	0.307
**Neonatal characteristics**			
Male, n (%)^b^	22 (55)	12 (60)	0.887
Birthweight, kg^a^	2.56 (1,56-3,15)	2.43 (1.69-3.16)	0.760
Apgar score <7 at 5 min, n (%)^b^	0 (0)	0 (0)	0.999
Birthweight <10^th^ percentile, n/N (%)^b^	5/40 (12.5)	1/20 (5)	0.653

Data described as the median (range).

a Mann-Whitney test.

b Fisher’s exact test.

BMI = body mass index; BP = blood pressure at time of diagnosis for preeclampsia group and at admission for control group; GA = gestational age; NA = not applicable; PE = preeclampsia.
